# Ecological Stoichiometric Relationships Among Wood‐Feeding Insects, Host Trees, and Soils in an Urban Tropical Ecosystem

**DOI:** 10.1002/ece3.73781

**Published:** 2026-06-02

**Authors:** Gabriel Adetoye Adedeji, Azuka Chinedum Egubogo, Adedapo Ayo Aiyeloja, Babatunde Solomon Ojelade, Israel Oluwaseyi David, Daniel Chukwuemeka Amaogu, Esther Tamunolgbani Nelson

**Affiliations:** ^1^ Department of Forestry and Wildlife Management University of Port Harcourt Port Harcourt Nigeria; ^2^ Department of Forestry, Faculty of Science, Engineering and Agriculture University of Venda Thohoyandou South Africa; ^3^ Department of Chemistry Federal University of Agriculture Abeokuta Nigeria; ^4^ Sustainable Infrastructure and Geoengineering Lab (SIGLab), Civil, Geological and Mining Engineering Department Polytechnique Montréal Montréal, Quebec Canada

**Keywords:** ecological stoichiometry, elemental ratios, nutrient dynamics, trophic mismatch, tropical urban ecosystem, wood‐feeding insects

## Abstract

Ecological stoichiometry aids in the understanding of nutrient imbalance in consumers and food resources, particularly in wood‐feeding systems, where the food source (plant tissues) is dominated by high levels of carbon and low levels of other critical elements. Nevertheless, the stoichiometric data for tropical terrestrial systems are underexplored, particularly for elements other than the classical C:N:P. This research investigated the stoichiometry of two wood‐feeding insects ( *Apate terebrans* and *Analeptes trifasciata*), their host trees (*Terminalia mantaly* and *Spondias mombin* ), and the adjacent soils of a tropical urban area. Trophic imbalances among insects, host tissues, and soils were evaluated by analysing elements N, P, Ca, Mg, Na, and C, and expressing them in stoichiometric ratios. There were notable differences in stoichiometry between the wood‐feeding insects and the host wood, particularly regarding N and P, affirming that the nutrient content of the woody substrate is low in comparison to the insects' elemental needs. *A. trifasciata* was more aligned with the host ratio of Ca and Mg than *A. terebrans* . The soil stoichiometry was associated with host plants, suggesting an indirect effect on insect nutrient dynamics via plant nutrient composition. These findings offer a starting point for the multi‐element stoichiometry of a tropical wood‐feeder–host–soil system and add to knowledge of nutrient limitations and trophic relationships in terrestrial ecosystems. The research provides a basis for subsequent experimental studies on the nutritional control and ecosystem nutrient cycling in wood‐feeding insect systems.

## Graphical abstract

In wood‐feeding insects, the presence of elemental composition suggests a potential nutritional deficiency. In this regard, the research aimed to examine the ecological stoichiometric mismatches between wood‐feeding insects and their tree hosts, analysing the components and stoichiometric mismatches of the forest ecosystem, as well as the role of soil. The study used biological stoichiometry principles to investigate the evolution of nutritional taste modalities in wood‐feeder insects, specifically *Apate terebrans* and Analeptes trifasciata, in relation to their feeding hosts, Terminalia mantaly and *Spondias mombin* , as well as the soil.

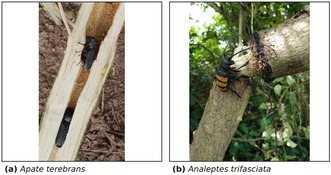

## 1 Introduction

Ecological stoichiometry is an approach to understanding how the balance of chemical elements in the environment regulates interactions among organisms and affects ecosystem functioning (Meunier et al. 2017; Welti et al. 2017). It is part of the ecosystem's organisation to recognise that living organisms require a limited number of elements in fairly constant proportions, whereas the environment/food sources contain these elements in varying amounts (Hessen et al. 2013; Meunier et al. 2017). Such a demand–supply imbalance in resources is especially common in terrestrial systems, where the structural tissues of plants contain much more carbon than nitrogen, phosphorus, and other important elements (Sardans et al. 2012; Sistla and Schimel 2012; Zhang et al. 2025). These imbalances lead to nutritional deficiencies in herbivores and detritivores and influence feeding patterns, nutrient cycling, and trophic levels in ecosystems.

Elemental homeostasis is a key concept in ecological stoichiometry; it describes how organisms retain internal elemental balance within a relatively constrained range, despite the changes in the elemental composition of their resources. This is especially crucial for understanding multi‐trophic linkages, as the level of homeostatic regulation determines how nutrient imbalances are transferred from soils to plants and then to consumers. Although early stoichiometric models often assumed relatively strict homeostasis (Wang et al. 2012), the degree of this assumption varies among organisms, elements, life stages, and ecological contexts. A more recent ionomic viewpoint emphasises that homeostasis should be assessed not only in the single C:N:P framework but also in terms of multiple elements, particularly calcium, magnesium, and sodium, which affect not only physiological regulation but also trophic interactions (Jeyasingh et al. 2017).

Siddiqa et al. (2023) indicated that wood, as a substrate, contains greater amounts of carbon, consisting of polysaccharides such as cellulose, hemicellulose, and lignin, and has low levels of nitrogen and phosphorus. As a result, wood‐feeding insects tend to exhibit greater stoichiometric imbalances between the substrates they consume and the elemental requirements of their bodies (Filipiak 2018; Lehenberger et al. 2021). It has been shown that insects have an internal stoichiometric ratio that is nutritionally unbalanced relative to the external ratios of the substrates, and in this case, it is higher in nitrogen and phosphorus than in the woody material (Filipiak and Weiner 2017; Filipiak 2018). In such systems where an element is limited, ecological stoichiometry is a key concept for studying the structure of interactions between consumers and resources.

Research on stoichiometry has primarily focused on aquatic systems and the C:N:P framework (Maranger et al. 2018; Tanioka et al. 2022), whereas available terrestrial datasets are limited in scope. Relatively little focus has been placed on calcium, magnesium, and sodium, even though these elements are critical for physiological, structural, and ionic regulatory processes in insects (Dow 2017; Mabelebele et al. 2023; Praveenkumar et al. 2024). To understand nutrient limitations in terrestrial food webs, especially in tropical ecosystems, stoichiometric studies must focus on other nutrients. This is particularly important, given that the chemistry of plants, decomposition processes, and nutrient cycles are likely to be quite different in tropical versus temperate ecosystems.

Girdlers and stemborers are wood‐feeding insects with significant ecological and economic importance as herbivores in rainforests and subtropical forests (Sreedevi et al. 2022; Dodds et al. 2023). *Apate terebrans* and *Analeptes trifasciata* are species that attack a wide range of woody hosts and are likely to affect forest systems in a significant way by impacting tree growth and survival, as well as nutrient redistribution. Despite the significance of these insects in the nutrient dynamics of forest systems, very little data are available on their elemental profiles and their relationships with host plants and soils. To understand the nutrient dynamics of these systems and the potential stoichiometric limitations affecting trophic interactions, it is important to examine and identify elemental variation in insects, host tissues, and surrounding soils (Filipiak 2018).

Soil nutrient availability adds a dimension to stoichiometric relationships in terrestrial systems. The elemental composition of soils affects plant nutrient absorption and, in turn, influences plant tissue chemistry, which then affects the quality of plant material available to herbivores (Zeng 2024). Therefore, the triad of insects, host plants, and soil provides an opportunity to understand nutrient dynamics across trophic levels and identify elemental imbalances within the ecosystem.

It has also been revealed by earlier studies that insect herbivore stoichiometry can track soil nutrient availability through plant‐mediated pathways, including soil nutrient tracking by desert insect herbivores and attenuation of C:N:P responses from soils to plants and insect herbivores in grassland systems (Schade et al. 2003; Hassan et al. 2021).

This study applies an ecological stoichiometric approach to characterise the elemental relationships among two wood‐feeding insects ( *Apate terebrans* and *Analeptes trifasciata*), their host trees (*Terminalia mantaly* and *Spondias mombin* ), and surrounding soils in an urban tropical environment. Specifically, the study aims to (i) quantify multi‐element stoichiometric ratios of insects, host tissues, and soils; (ii) evaluate the extent of stoichiometric mismatch between wood‐feeding insects and their feeding substrates; and (iii) examine the associations between soil stoichiometry and the elemental composition of host plants and insects. By providing baseline data from an understudied terrestrial system and incorporating elements beyond the traditional C:N:P framework, this work improves understanding of nutrient constraints and trophic relationships in tropical ecosystems and establishes a foundation for future experimental investigations of nutritional regulation in wood‐feeding insect systems.

## 2 Methodology

### 2.1 Study Area and Study System

**FIGURE 1:**
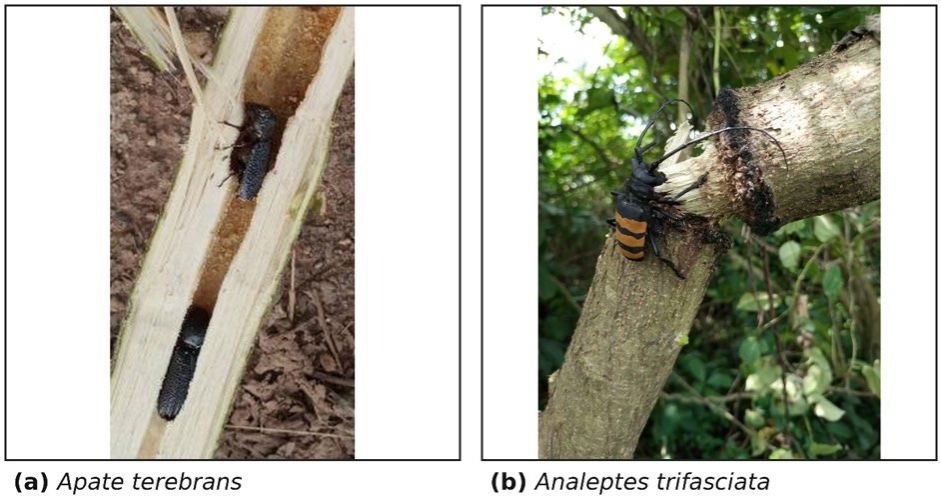
Representative wood‐feeding insects examined in this study.

The study was conducted within an urban tropical environment in the University of Port Harcourt, Rivers State, Nigeria, located at Latitude 4°53′25″ and 4° 54′ 35″ N and Longitude 6°54′25″ and 6°55′55″ E. The study area is characterised by managed landscapes where ornamental and fruit tree species are commonly established. The study focused on two wood‐feeding insect species, *Apate terebrans* (Coleoptera: Bostrichidae) and *Analeptes trifasciata* (Coleoptera: Cerambycidae), which are known to infest the woody tissues of several tropical tree species (Figure 1). On the basis of visible signs of infestation and active wood‐feeding individuals, two frequently infested host trees, *Terminalia mantaly* and *Spondias mombin* , were selected.

Both insect species target woody tissues, and their larvae develop in the stems/woody sections of the host plants. Sampling focused on active feeding sites to confine elemental comparisons to the basal feeding substrate. Soil samples were collected immediately around each host tree to capture the nutritional context in which the host plants were absorbing nutrients. Sampling was conducted in a single event owing to similar environmental conditions, which would minimise temporal variability in elemental concentrations.

### 2.2 Sample Collection and Preparation

Wood‐feeding insects were collected directly from actively infested host trees. A total of four individuals of *Apate terebrans* were collected, from four different *Terminalia mantaly* trees, and also four individuals of *Analeptes trifasciata* were collected from four different *Spondias mombin* trees. From each infested tree, four insect individuals were collected from the feeding galleries or damaged woody tissues. Only adults were used for elemental analysis to minimise variation associated with insect development stage, because insect stoichiometry can vary between larvae and adults. Collected insects were stored in ethanol and oven‐dried at 55°C before analysis. Dried insect samples were ground and homogenised to achieve uniform consistency for elemental analysis.

Host plant samples were obtained from the same individual trees from which insects were collected. The sampled plant material comprised stem wood and bark. For each tree, tissue samples were taken at breast height using an increment borer. Plant materials were air‐dried, ground, and sieved to obtain a uniform particle size for analysis.

Soil samples were collected from the rooting zone of each sampled host tree after removal of surface litter. For each tree, four soil cores were collected at a distance of 0.5 m from the tree trunk and at a depth of 0–15 cm. Where multiple cores were collected around the same tree, they were combined into one composite soil sample per tree before laboratory preparation. The samples were air‐dried, homogenised, and sieved to eliminate stones, roots, and other materials. All prepared samples were kept in airtight containers prior to laboratory analysis.

### 2.3 Elemental Analysis

Using standard procedures for elemental analysis of soils, plants, and insect tissues, samples were prepared and analysed for nitrogen (N), phosphorus (P), calcium (Ca), magnesium (Mg), sodium (Na), and organic carbon (C) (Page et al. 1982; Nelson and Sommers 1996; AOAC 2000). Spectrophotometric methods were used for the extraction and determination of nitrogen and phosphorus; flame photometry was used for sodium; ethylene‐diamine‐tetra‐acetic acid titration was used for calcium and magnesium; and the Walkley–Black method was used for organic carbon (Nelson and Sommers 1996; Sikora and Moore 2014). Environmental sample quality control standards were adhered to during analyses. Stoichiometric calculations were performed after elemental concentrations were expressed on a mass basis.

### 2.4 Stoichiometric Calculations

In line with ecological stoichiometry practices, elemental data were converted to carbon‐based ratios, as carbon is the principal structural component of woody tissues. The primary stoichiometric ratios calculated for insects, host tissues, and soils were C:N, C:P, C:Ca, C:Mg, and C:Na, following the general ecological stoichiometry approach of expressing nutrient availability relative to carbon content (Sterner and Elser 2002; Filipiak and Weiner 2014).

Trophic stoichiometric ratios (TSR) were then calculated to evaluate elemental imbalance between wood‐feeding insects and their host substrate. For each element X, TSR was calculated as follows: TSR_X = (C:X ratio of host tissue)/(C:X ratio of insect tissue), where X represents N, P, Ca, Mg, or Na. Thus, the values in Table 2 represent ratios of the carbon‐normalised elemental composition between host and insect tissues, rather than direct concentration ratios. TSR values close to 1 indicate strong stoichiometric similarity between consumers and their resources, whereas values greater than 1 indicate that the host tissue has a higher C:X ratio than the insect and is therefore relatively poorer in element X compared with consumer demand (Sterner and Elser 2002; Filipiak and Weiner 2014; Filipiak 2018).

### 2.5 Statistical Analysis

The differences in elemental ratios between insects, host tissues, and soils were analysed using analysis of variance, and post hoc comparisons were applied when appropriate. Relationships among the soil, the host plant, and wood‐feeding insects were assessed using Pearson correlation analyses of soil stoichiometric ratios and host plant and insect ratios, enabling evaluation of possible soil–plant–consumer linkages.

Because of the multivariate relationships among elemental ratios, principal component analysis (PCA) was performed to examine these relationships and identify the dominant gradients underlying variations in elemental ratios among insects, host tissues, and soil samples. All statistical analyses were performed using standard statistical packages, and significance was assessed at the 0.05 probability level.

## 3 Results

### 3.1 Elemental Concentrations and Stoichiometric Composition of Wood‐Feeding Insects, Host Tissues, and Soils

Table 1 shows differences in elemental composition, including concentrations and ratios, among wood‐feeding insects, their host tissues, and soils. The insects *Apate terebrans* and *Analeptes trifasciata* had tissue nitrogen levels higher than those of host wood and soil samples. *A. trifasciata* and *A. terebrans* have tissue Phosphorus levels higher than their host wood tissue Phosphorus levels; *A. trifasciata* again shows a higher concentration of phosphorus in its tissue than *A. terebrans* . These observations align with earlier studies, which reported higher levels of Nitrogen and Phosphorus in insect tissues than in woody substrates, because of metabolic and structural requirements (Elser, Fagan, et al. 2000; Filipiak and Weiner 2014).

**TABLE 1:** Mean comparison of elemental composition in selected wood‐feeders and their hosts' wood and stem.

Wood‐feeders/Host	N (%)	P (mg/kg)	Ca (mg/kg)	Mg (mg/kg)	Na (mg/kg)	Org. C (%)
*A. terebrans*	2.13 ± 0.08^a^	88.63 ± 5.12^b^	4400.88 ± 700.65^a^	27.55 ± 1.51^c^	2561.39 ± 54.89^a^	15.16 ± 0.10^d^
*A. trifasciata*	0.45 ± 0.01^b^	434.64 ± 6.70^a^	39.52 ± 2.68^d^	31.15 ± 0.33^a^	829.67 ± 8.50^c^	39.59 ± 1.71^a^
*T. mantaly* (Wood)	0.03 ± 0.00^de^	31.54 ± 0.62^e^	54.91 ± 2.13^d^	29.22 ± 0.65^b^	279.68 ± 23.24^e^	39.95 ± 2.24^a^
*S. mombin* (Wood)	0.03 ± 0.00^de^	20.98 ± 1.32^f^	85.42 ± 1.34^d^	30.29 ± 0.65^ab^	350.33 ± 1.83^d^	16.17 ± 0.31^d^
*T. mantaly* (Stem)	0.07 ± 0.00^d^	62.28 ± 1.43^c^	353.04 ± 10.75^cd^	31.72 ± 0.45^a^	891.28 ± 11.31^b^	18.07 ± 0.26^c^
*S. mombin* (Stem)	0.23 ± 0.00^c^	21.40 ± 0.47^f^	567.83 ± 14.32^c^	30.17 ± 1.27^ab^	318.15 ± 2.92^d^	20.82 ± 0.37^b^
*T. mantaly* (Soil)	0.02 ± 0.00^e^	41.74 ± 0.67^d^	1905.40 ± 9.07^b^	27.52 ± 0.68^c^	30.59 ± 3.74^f^	1.96 ± 0.07^e^
*S. mombin* (Soil)	0.04 ± 0.00^de^	29.89 ± 1.91^e^	54.35 ± 1.39^d^	27.66 ± 0.57^c^	40.27 ± 1.31^f^	1.87 ± 0.13^e^

*Note:* Means with the same superscripts are not significantly different within the column at *p* < 0.05.

Calcium content varied widely across different sample types. *A. terebrans* recorded the largest calcium concentration (4400.88 mg kg^−1^), which is considerably larger than the calcium concentrations found in the host tissues and soils. *A. trifasciata* has a calcium concentration of (39.52 mg kg^−1^), which is very small in comparison. The calcium concentration in the host stems ranged from 353.04 to 567.83 mg kg^−1^, and the soil for *T. mantaly* had a higher‐than‐usual calcium concentration (1905.40 mg kg^−1^). The magnesium concentrations in soil and tissues for different types of insects are quite similar (27.52 mg kg^−1^), whereas those for sodium are not. Sodium concentrations in *A. terebrans* are the highest (2561.39 mg kg^−1^), and the soil concentration is the lowest (30.59–40.27 mg kg^−1^) (Table 1). The highest soil organic carbon concentration was in the host tree stems, which also had the lowest concentration among soil types, reflecting the high organic carbon content in woody biomass (Pettersen 1984).

With regards to the stoichiometric ratios concerning carbon, insects show a consistently lower ratio of C:N and C:P when compared to the host wood and stem tissues. This observation implies an increased relative presence of nitrogen and phosphorus in insect biomass when compared to the feeding substrates (Table 1). This finding is consistent with previously established expectations in stoichiometry for xylophagous systems, in which consumers possess greater tissue nutrient levels than the carbon‐rich food plant (Sterner and Elser 2002; Filipiak 2016). The C:Ca, C:Mg, and C:Na ratios reflected an interspecific elemental relationship, where *A. trifasciata* showed a closer relationship with host tissues regarding calcium and magnesium than *A. terebrans* .

### 3.2 Trophic Stoichiometric Mismatch Between Wood‐Feeders and Feeding Hosts

TSRs show a large imbalance between the elemental composition of insects and the elemental composition of the wood hosts (Table 2). *A. terebrans* compared to *T. mantaly* wood exhibited significant mismatches for nitrogen (182.90), calcium (210.80), and sodium (24.38), and moderate mismatches for phosphorus and magnesium (7.39 and 2.48, respectively). These results demonstrate the classic imbalance in elemental requirements for insect biomass and the low nutrients provided by woody substrates (Elser, Fagan, et al. 2000; Filipiak and Weiner 2014). In comparison with the host stem tissues, the mismatches were lower but still pronounced for nitrogen (35.83) and calcium (14.82), whereas phosphorus (1.70), magnesium (1.04), and sodium (3.42) showed smaller deviations from the stochiometric balance.

**TABLE 2:** Trophic stoichiometry ratio averages of selected wood‐feeders.

Host	Wood‐feeder	N	P	Ca	Mg	Na
*Wood*						
*T. mantaly*	*A. terebrans*	182.90	7.39	210.80	2.48	24.38
*S. mombin*	*A. trifasciata*	6.91	8.49	0.19	0.42	0.97
*Stem*						
*T. mantaly*	*A. terebrans*	35.83	1.70	14.82	1.04	3.42
*S. mombin*	*A. trifasciata*	1.05	10.70	0.04	0.55	1.37
*Pooled*						
	*A. terebrans*	109.37	4.55	112.81	1.76	13.90
	*A. trifasciata*	3.98	9.60	0.11	0.48	1.17

On the other hand, *A. trifasciata* exhibited smaller mismatches with respect to its host wood, with calcium, magnesium, and sodium ratios of 0.19, 0.42, and 0.97, respectively, indicating greater stoichiometric balance for these elements (Table 2). However, nitrogen (6.91) and phosphorus (8.49) were still poorly matched to the host wood. Comparisons with the stem tissues showed closer balance for calcium (0.04) and magnesium (0.55), whereas phosphorus remained very strongly mismatched (10.70). The overall pattern of very strong mismatches in nitrogen and phosphorus in host tissues confirmed the general stoichiometric constraints on wood‐feeding organisms (Filipiak 2016).

### 3.3 Relationships Between Soil Stoichiometry and Insect–Host Stoichiometry

Correlation analysis showed some strong relationships between soil stoichiometric ratios and the elemental composition of the host plants (Tables 3 and 4). In the *T. mantaly* system, soil C:N ratios showed strong positive correlations with host wood C:N (*r* = 0.99), C:Mg (*r* = 1.00), and C:Na (*r* = 1.00) ratios (Table 3). This shows plant stoichiometry is influenced by soil nutrient conditions. Soil–plant relationships where soil nutrient availability controls the chemistry of plant tissues are common in terrestrial ecosystems and affect herbivore nutrition in an indirect way (Giesler et al. 2004; Hartley and Jones 2008). A strong negative relationship was found between soil C:Na and insect C:Mg (*r* = −0.99), indicating opposite elemental gradients between the soil and the insects.

**TABLE 3:** Correlation between soil stoichiometry traits and stoichiometry of *A. terebrans* and *T. mantaly* wood and stem.

	Soil C:N	Soil C:P	Soil C:Ca	Soil C:Mg	Soil C:Na
Insect C:N	−0.99	−0.11	−0.67	−0.47	1.00
Insect C:P	1.00	0.30	0.80	0.63	−0.96
Insect C:Ca	0.81	−0.39	0.22	−0.03	−0.91
Insect C:Mg	0.99	0.09	0.65	0.44	−0.99*
Insect C:Na	−0.86	−0.70	−0.99	−0.91	0.74
Wood C:N	0.99*	0.17	0.71	0.51	−0.99
Wood C:P	0.95	−0.09	0.51	0.28	−0.99
Wood C:Ca	0.95	0.51	0.92	0.79	−0.88
Wood C:Mg	1.00*	0.20	0.73	0.54	−0.99
Wood C:Na	1.00**	0.22	0.75	0.56	−0.98
Stem C:N	−0.65	−0.89	−0.99	−0.99	0.49
Stem C:P	−0.99	−0.38	−0.85	−0.69	0.94
Stem C:Ca	0.75	−0.48	0.12	−0.13	−0.86
Stem C:Mg	−0.93	−0.56	−0.94	−0.82	0.85
Stem C:Na	−0.95	−0.51	−0.91	−0.78	0.88

* Correlation is significant at the 0.05 level.

** Correlation is significant at the 0.01 level.

**TABLE 4:** Correlation between soil stoichiometry traits and stoichiometry of *A. trifasciata* and *S. mombin* wood and stem.

	Soil C:N	Soil C:P	Soil C:Ca	Soil C:Mg	Soil C:Na
Insect C:N	0.77	0.95	0.99	0.99*	0.93
Insect C:P	0.94	1.00*	0.90	0.95	0.74
Insect C:Ca	−0.95	−0.99*	−0.89	−0.95	−0.73
Insect C:Mg	0.95	0.99*	0.89	0.94	0.72
Insect C:Na	0.95	0.99*	0.90	0.95	0.74
Wood C:N	0.76	0.94	1.00	0.99*	0.93
Wood C:P	−0.95	−0.77	−0.44	−0.57	−0.17
Wood C:Ca	0.84	0.98	0.98	0.99*	0.88
Wood C:Mg	0.99*	0.95	0.75	0.84	0.53
Wood C:Na	0.96	1.00	0.88	0.94	0.71
Stem C:N	0.43	0.07	−0.35	−0.21	−0.60
Stem C:P	−1.00	−0.96	−0.77	−0.85	−0.56
Stem C:Ca	−0.91	−0.99*	−0.94	−0.98	−0.80
Stem C:Mg	−0.97	−0.99	−0.86	−0.92	−0.68
Stem C:Na	−0.62	−0.86	−0.99	−0.97	−0.99

* Correlation is significant at the 0.05 level.

In the *S. mombin* system, soil C:P ratios showed strong positive correlations with insect C:P (*r* = 1.00), C:Mg (*r* = 0.99), and C:Na (*r* = 0.99), and strong negative correlations with insect C:Ca (*r* = −0.99) and stem C:P ratios (Table 4). In general, soil stoichiometry indicated stronger correlations with tissue ratios of host plants compared to tissue ratios of insects, reinforcing the assumption that the effects of soil nutrients operate through plant‐mediated pathways as opposed to plant‐insect pathways.

### 3.4 Multivariate Stoichiometric Relationships Among Insects, Hosts, and Soils

**FIGURE 2:**
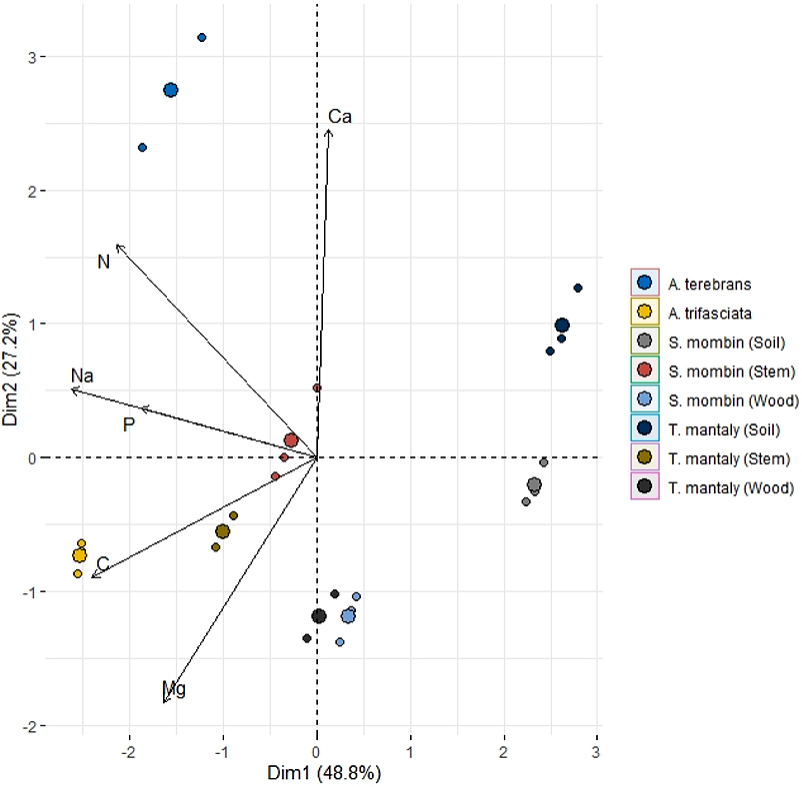
Multivariate analysis of stoichiometric relations between wood‐feeders and host on the basis of the elements studied.

PCA summarised multi‐element stoichiometric relationships among insects, host tissues, and soils (Figure 2). The first two principal components accounted for 76.0% total variance in the dataset. Although the first principal component (PC1) accounted for 48.8%, the second principal component (PC2) accounted for 27.2%.

PC1 was mainly linked to sodium, phosphorus, and carbon ratios, and along this gradient, the insect samples were separated from the wood and soil samples. It was mainly PC2 that was influenced by calcium‐to‐magnesium ratios and distinguished *A. terebrans* from *A. trifasciata* , reflecting the differences in chemical composition between the two species.

The clustering of insects, host tissues, and soils within multivariate space suggests clear stoichiometric differentiation within trophic components. In PC2, *A. terebrans* exhibited strong positive loading, which can be attributed to increased relative calcium and magnesium concentration, whereas *A. trifasciata* was more strongly associated with PC1, which can be attributed to the carbon and phosphorus ratios. Additionally, the greater separation of soil samples along PC1 indicated their contribution to the variability in sodium and phosphorus within the system. The separation of trophic components along multivariate axes can be explained by nutrient relationships in ecological stoichiometry, which proposes that several elements work together to drive trophic differentiation and ecosystem function (Sterner and Elser 2002; Branco et al. 2018). The multivariate patterns indicate elemental contrasts among insects, host plants, and soils and demonstrate the role of multi‐element stoichiometry in driving the ecosystem's nutrient dynamics.

## 4 Discussion

This study provides a multi‐element ecological stoichiometric characterisation of two wood‐feeding insects, their host trees, and surrounding soils within a tropical urban environment. When elemental composition is expressed in terms of ratios relative to carbon, there is a clear stoichiometric difference between the consumers and the woody substrates, suggesting the elemental imbalance is a key structuring feature of wood‐feeding systems. Ecological stoichiometry is the study of the balance of multiple chemical elements in ecological communities. In this discipline, a major focus of inquiry is the limited range of elemental proportions required by organisms (as opposed to the wide range of proportions that can be present in resource supplies) and the effects on imbalances in the trophic structures of ecosystems—what and how organisms consume, and the resultant effects on the functioning of the ecosystems (Sterner and Elser 2002; Elser, Sterner, et al. 2000). Primary data on the stoichiometry of tropical terrestrial ecosystems, particularly on elements outside C:N:P, are lacking, and the current study offers a starting point to address this gap (Filipiak and Weiner 2014; Sperfeld et al. 2017). A major finding in this study is the pronounced elemental imbalance, particularly in N and P, between the insects and the host wood. The wood tissues exhibited a higher C:N ratio than the insect biomass. Therefore, woody substrates are deficient in the elements required by insects in greater proportions. Structural wood components contain large amounts of cellulose, hemicellulose, and lignin, which are poor in nitrogen and phosphorus (Pettersen 1984; Meerts 2002). Similar trends have been reported in xylophagous systems, where insects usually hold a greater concentration of nitrogen and phosphorus than the substrates they feed on (Filipiak and Weiner 2014; Filipiak 2016). The differences in C:N and C:P ratios are therefore a reflection of the basic trophic imbalance rather than a species‐specific anomaly, and are in line with the prevailing hypothesis that wood‐feeding organisms are nutrient‐limited when compared to the carbon‐rich diet (Elser, Fagan, et al. 2000).

The marked differences between insect tissues and woody host materials can also be interpreted through the lens of elemental homeostasis. Insects are expected to maintain relatively constrained internal elemental composition compared with the highly variable and nutrient‐poor woody substrates on which they feed. Therefore, the lower C:N and C:P ratios in insect tissues, and the enrichment of elements such as nitrogen and phosphorus relative to host wood, suggest not only trophic mismatch but also the outcome of physiological regulation that allows consumers to sustain body composition despite resource imbalance. However, the present results should not be interpreted as evidence of strict homeostasis. As argued by Wang et al. (2012), strict homeostasis is a simplifying assumption and may not hold equally across all organisms or elements. The species‐specific contrasts observed here, particularly for calcium, magnesium, and sodium, are consistent with a more flexible, multi‐element view of homeostasis in which different elements may be regulated to different degrees (Jeyasingh et al. 2017).

Differences between the two insect species further highlight the importance of multi‐element perspectives in ecological stoichiometry. *Apate terebrans* exhibited strong mismatches with host tissues for calcium and sodium in addition to nitrogen, whereas *Analeptes trifasciata* showed closer alignment with host tissues for calcium and magnesium. These differences do not necessarily indicate active optimisation of feeding behaviour but rather reflect species‐specific physiological composition and nutrient demands. Calcium plays important structural and physiological roles in insects, including cuticular stability and ion regulation, and variation in calcium‐related ratios may, therefore, arise from differences in body composition rather than nutrient substitution among elements. The close correspondence between *A. trifasciata* and its host for calcium and magnesium indicates reduced elemental imbalance rather than evidence of selective regulation, consistent with stoichiometric theory emphasising physiological constraints on elemental composition (Sterner and Elser 2002; Sperfeld et al. 2017).

One possible explanation for the large difference in TSR between the two insect species is that they differ in their feeding niches, host‐tissue use, and physiological elemental demands. *A. terebrans* may exploit woody tissues that are more carbon‐rich and relatively poorer in several mineral nutrients, while also maintaining comparatively high tissue requirements for elements such as nitrogen, calcium, and sodium; this combination would generate high TSR values. In contrast, *A. trifasciata* may feed on host tissues whose elemental composition is closer to its own, or it may have lower relative requirements for some elements, resulting in lower TSR values. This hypothesis remains speculative because feeding microhabitat, assimilation efficiency, developmental stage, and microbial contributions to nutrition were not experimentally tested, but it provides a useful direction for future work on species‐specific nutrient regulation in wood‐feeding insects.

The observed relationships among soil, host plants, and insects highlight the soil nutrient status in an indirect role in the formation of trophic stoichiometry. The strong correlation between soil and stoichiometric ratios of host plants suggests that soil nutrient availability affects the elemental composition of plants, which in turn determines the amount of nutrients that the herbivore can assimilate. The same plant‐mediated pathways that soil nutrient dynamics have on herbivore nutrition have also been recognised in terrestrial ecosystems. For instance, the alteration of soil nutrient availability in plant tissue chemistry affects herbivore performance and nutrient cycling (Hartley and Jones 2008; Giesler et al. 2004). The observed weak and inconsistent correlation between soil and insect stoichiometry in this study suggests that insects have maintained their elemental composition within a relatively narrow range, regardless of the resource quality. This finding aligns with the idea of stoichiometric regulation at the organism level (Sterner and Elser 2002). These results are consistent with previous evidence that soil nutrient signals can propagate upward through plant tissues to insect herbivores, although the strength of this linkage may weaken across trophic levels (Schade et al. 2003; Hassan et al. 2021).

The patterns seen in multivariate PCA confirm the existence of stoichiometric differentiation among insects, host tissues, and soils. The positioning of the different sample types along the different ranges of phosphorus, sodium, calcium, and magnesium demonstrates an elemental synergy in influencing different trophic levels in the system. Such synergistic interaction of multiple elements corresponds with the emerging trends in ecological stoichiometry studies showing that the interaction of different elements, rather than a single element, is what drives the dynamics of an ecosystem (Branco et al. 2018; Sperfeld et al. 2017), The distinct separation of soils, plant tissues and insects shows the elemental poverty spanning across different trophic levels and shows that multiple elements, rather than a single element, should be looked at to increase the understanding of the ecological dynamics in a terrestrial ecosystem. The interpretation of the results must be done within the boundaries provided by the field‐based characterisation. It should be noted that the field‐based characterisation was achieved through the identification of the misalignments, which, although consistent, aligned with the theoretical expectations. However, this study does not experimentally evaluate mechanisms such as compensatory feeding, physiological regulation, or homeostatic control. Other studies have shown that such mechanisms are the result of deliberate, experimental, and controlled studies (Simpson and Raubenheimer 2012). Consequently, the present study provides the basic, foundational understanding of the elemental relations that can be used to plan future experimental studies about how wood‐feeding insects respond to nutritionally unbalanced substrates. Research should also address seasonal variation, developmental stage differences, and variation among plant tissues that may influence stoichiometric relationships.

This study is the first of its kind to show that systems of wood‐feeding insects in tropical regions are characterised by marked multi‐element stoichiometric contrasts between consumers and woody substrates, where host soil nutrient status also affects these relationships indirectly. By going beyond traditional C:N:P ratios and including calcium, magnesium, and sodium, this study broadens understanding of nutrient limitation and trophic dynamics in terrestrial systems and identifies the wood‐feeding insect community as a system for studying the ecological and functional effects of elemental imbalance.

## 5 Conclusion

This ecological baseline study characterises two species of wood‐feeding insects, their host trees, and the associated soil within a tropical urban ecosystem. This study records the stoichiometric dissociation between insect consumers and woody substrates. Whereas host wood is enriched in carbon (C), insect tissue is enriched in nitrogen (N) and phosphorus (P). This result shows the stoichiometric imbalance in elemental composition in wood‐feeding systems. It illustrates the elemental dissociation in the evolutionary development of wood‐feeding systems and the imbalance in insect biomass needs. Species‐specific variations demonstrate the necessity of going beyond the classical C:N:P triad. *Apate terebrans* illustrated considerable deviation for N, Ca, and Na, whereas *Analeptes trifasciata* had host tissue‐like Ca and Mg. Instead of active regulation, a physiological demand for these elements and their composition formed the variance, demonstrating the extent of biological control in trophic stoichiometry.

This study concludes that soil nutrient composition is indicative of the stoichiometric relations between insects and their hosts, as well as of the elemental composition of the plants. The soil and host plant stoichiometry illustrated the soil conditions made available to wood‐eating insects and the integrated roles of soil, plants, and consumers in the elemental cycles of terrestrial ecosystems.

The specific differences in stoichiometry between insects, host tissues, and soils, as shown by multivariate analysis, show that different elements affect the trophic linkages in the system. From a multidimensional perspective, the inclusion of calcium, magnesium, sodium, and together with nitrogen and phosphorus, the nutrient constraints in terrestrial ecosystems reinforces the understanding of nutrient limitation. It underscores the multiplicity of factors necessary in the relations among ecosystems.

The study provides empirical evidence that describes the first step in creating a baseline to evaluate mechanistic processes. To understand how wood‐feeding insects respond to different nutritional variabilities in a given environment, there needs to be more detailed methodological approaches, including seasonal sampling, targeted studies in controlled laboratory environments, and studies focusing on specific developmental instars of the insects. Nonetheless, this study is a significant contribution to our knowledge of the elemental compositions of the systems of tropical wood‐feeding insects and of the nutrient relationships in terrestrial ecosystems.

## Author Contributions

**Gabriel Adetoye Adedeji:** conceptualization (equal), data curation (equal), formal analysis (equal), investigation (equal), methodology (equal), project administration (equal), supervision (equal), writing – original draft (equal), writing – review and editing (equal). **Azuka Chinedum Egubogo:** conceptualization (equal), data curation (equal), investigation (equal), methodology (equal), writing – original draft (equal), writing – review and editing (equal). **Adedapo Ayo Aiyeloja:** data curation (equal), formal analysis (equal), supervision (equal), writing – review and editing (equal). **Babatunde Solomon Ojelade:** conceptualization (equal), data curation (equal), formal analysis (equal), writing – original draft (equal), writing – review and editing (equal). **Israel Oluwaseyi David:** data curation (equal), formal analysis (equal), methodology (equal), writing – review and editing (equal). **Daniel Chukwuemeka Amaogu:** conceptualization (equal), formal analysis (equal), methodology (equal), writing – review and editing (equal). **Esther Tamunolgbani Nelson:** conceptualization (equal), data curation (equal), formal analysis (equal), methodology (equal), writing – original draft (equal), writing – review and editing (equal).

## Funding

The authors have nothing to report.

## Conflicts of Interest

The authors declare no conflicts of interest.

## Data Availability

The data that support the findings of this study are available in the Supporting Information of this article.
